# Application of nano-hydroxyapatite/chitosan scaffolds on rat calvarial critical-sized defects: A pilot study

**DOI:** 10.4317/medoral.22455

**Published:** 2018-09-28

**Authors:** Emmanouil Chatzipetros, Panos Christopoulos, Catherine Donta, Konstantinos I. Tosios, Evangelos Tsiambas, Dimitris Tsiourvas, Eleni-Marina Kalogirou, Kostas Tsiklakis

**Affiliations:** 1 DDS, MSc, PhD Candidate, Department of Oral Diagnosis and Radiology, Faculty of Dentistry, National and Kapodistrian University of Athens, Athens, Greece; 2 DDS, PhD, Assistant Professor, Department of Oral and Maxillofacial Surgery, Faculty of Dentistry, National and Kapodistrian University of Athens, Athens, Greece; 3 DDS, PhD, Associate Professor, Department of Oral Diagnosis and Radiology, Faculty of Dentistry, National and Kapodistrian University of Athens, Athens, Greece; 4 DDS, PhD, Assistant Professor, Department of Oral Medicine and Pathology, Faculty of Dentistry, National and Kapodistrian University of Athens, Athens, Greece; 5 MD, MSc, PhD, Lecturer, Department of Immunochistochemistry and Molecular Biology, 401 Military Hospital of Athens, Athens, Greece; 6 PhD, Institute of Nanoscience and Nanotechnology, National Centre for Scientific Research “Demokritos”, Athens, Greece; 7 DDS, MSc, PhD Candidate, Department of Oral Medicine and Pathology, Faculty of Dentistry, National and Kapodistrian University of Athens, Athens, Greece; 8 DDS, MSc, PhD, Professor, Department of Oral Diagnosis and Radiology, Faculty of Dentistry, National and Kapodistrian University of Athens, Athens, Greece

## Abstract

**Background:**

The purpose of this pilot study was to evaluate for the first time the effect of 75/25 w/w nano-Hydroxyapatite/Chitosan (nHAp/CS) scaffolds on Guided Bone Regeneration (GBR) in rat calvarial critical-sized defects (CSDs).

**Material and Methods:**

Six adult Sprague Dawley rats, 3 males and 3 females, were used. Two CSDs, full thickness and 5mm in diameter, were trephined in both sides of the parietal bone. The right CSD was filled with nHAp/CS scaffold, while the left CSD remained empty, as the control group. The wound was sutured in layers. Rats were euthanized with diethyl ether inhalation at 2, 4 and 8 weeks after surgical procedure. Histological and histomorphometric analysis was performed within distinct regions of interest (ROI): the lateral area inward of the middle sagittal seam; the lateral area outward of the middle sagittal seam and the central area.

**Results:**

The mean surface of newly formed bone (in μm2) in the lateral area inward of the middle sagittal seam of all rats was significantly higher (*P*=0.039) in the experimental group (91733.00±38855.60) than the control group (46762.17±25507.97). The NOex-c, defined as total number of osteocytes (OST) in newly formed bone surface in experimental group [experimental OST] minus the total number of osteocytes in newly formed bone surface in control group [control OST], was significantly greater (*P*=0.029) at 4th week post-surgery. Within the experimental group, a statistically significant increase (*P*=0.042) in the surface of newly formed bone was noticed in rats euthanized in 4th week compared with rats euthanized in 2nd week after surgery in the lateral area inward of the middle sagittal seam.

**Conclusions:**

The results of this study suggest that 75/25 w/w nHAp/CS scaffolds should be considered as a suitable biomaterial for GBR.

** Key words:**Hydroxyapatite, chitosan, critical sized defect, rats, bone regeneration.

## Introduction

Various bone graft materials provide an osteoconductive matrix that enhances bone formation in hard tissue critical-sized defects (CSDs) (1). For successful application, those bone graft materials should possess characteristics close to those of natural bone, as well as a pore structure that would allow the migration of osteoblasts and promote their growth and proliferation (2). Given that bone is a highly specialized composite material consisting of hydroxyapatite embedded in a polymeric matrix of collagen and various non-collageneous proteins, the current research efforts are mainly focused on the development of composite hydroxyapatite/polymer porous scaffolds. Polymers of natural origin such as gelatin, collagen, proteoglycans, alginates and chitosan (CS) have been employed in the development of such scaffolds (3,4). Among them, CS promotes osteoconduction, ability to produce porous structures and cell ingrowth, antibacterial nature and minimal foreign body reaction. In addition, CS has structural similarity to glycosaminoglycans (5,6) and permits the incorporation of bioceramics, such as hydroxyapatite (7).

Synthetic hydroxyapatite (HAp) has been used in Guided Bone Regeneration (GBR) as a bone substitute, as it is very similar to biogenic apatite (8) and has excellent biocompatibility, non-toxicity, non-immunogenic behavior, and osteoconductive ability (9,10). HAp particles suitable for bone tissue regeneration should have narrow crystal size distribution, high purity and high specific surface area (11). In recent studies, biomaterials have been used in GBR as porous scaffolds, usually consisting of nano-Hydroxyapatite (nHAp) and chitosan (12-16). Nano-hydroxyapatite/Chitosan (nHAp/CS) is a composite, inorganic/organic scaffold for GBR that combines improved mechanical properties and the desired properties of the ideal biomaterial, e.g. hemocompatibility, biocompatibility and osteoconductivity equivalent to natural bone (1,17). Due to its controlled porous structure, the scaffold promotes cell proliferation, growth and migration, thus, representing a promising tool in tissue engineering applications (17).

The study of GBR often involves experimental *in vivo* models. Rat calvaria is one of the most used pre-clinical settings for the testing of osseous graft materials. Rat calvarial defect model is relatively inexpensive and provides adequate anatomical access and easy surgical procedure (18).

Previously, the biological behavior of varying weight (w) proportions of nHAp/CS, i.e. 20/80 w/w, 30/70 w/w, 50/50 w/w and 80/20 w/w, in rats has been investigated. The nHAp/CS composites were biocompatible and biodegradable, with sufficient elasticity and flexibility (13). It should be noted that an increased hydroxyapatite concentration exceeding 80% has been reported to result in friable scaffolds *in vitro* (17). In contrast, scaffolds with a nHAp/CS concentration of 75/25 w/w have shown increased physico-mechanical characteristics (17). Based on these results of the *in vitro* study of Tsiourvas *et al.* (17), we hypothesized that nHAp/CS 75/25 w/w may show also increased physico-mechanical and osteoconductive characteristics when applied to an *in vivo* rat calvarial model.

The objectives of this pilot study were to evaluate for the first time the effect of 75/25 w/w nHAp/CS scaffolds on new bone formation and to estimate the biological behavior of nHAp/CS scaffolds in rat calvarial critical-sized defects (CSDs). The new bone formation and the total number of osteocytes in new bone surface were analyzed comparing the CSDs loaded with nHAp/CS scaffolds with empty CSDs.

## Material and Methods

This was an *in vivo* experimental pilot study, since there is no background in the use of the biomaterial nHAp/CS 75/25 w/w.

- Fabrication of Chitosan/nano-Hydroxyapatite compo-site scaffolds

Nanoparticles of hydroxyapatite (nHAp) were synthesized according to a previous work (19) in the presence of hyperbranched polyethylene imine (Lupasol G100, BASF, Greece), in order to regulate the size and morphology of hydroxyapatite crystals. This method affords hydroxyapatite nanoparticles having a monodisperse size distribution that can be utilized for the preparation of water colloidal dispersions with concentrations up to 9% w/w, which exhibit excellent stability, remaining stable even after storage (50C) for more than one year. These features are essential for the preparation of well-dispersed HAp/CS composites.

Composite porous scaffolds were developed by preparing a 3% w/w chitosan (Sigma-Aldrich, high molecular weight, deacetylation degree ≥75.0%, Milwaukee, WI, USA) solution in aqueous acetic acid (1.5% w/w) and adding nHAp to a final HAp/CS weight ratio of 75/25 (17). The resulting thick slurry was thoroughly mixed and molded in glass tubes (5mm i.d.) that were subsequently frozen at -25oC and lyophilized. After lyophilization the resulting nHAp/CS cylindrical porous scaffolds (5mm in diameter) were cut to disks of 1mm thickness, ethanol sterilized and extensively washed with sterile phosphate buffer saline inside a laminar flow cabinet. Porosity and total pore volume were found to be 85±2% and 5.0±0.5 ml/g, respectively, as established by determining the volume of liquid infused in the pores of dried scaffolds (20).

- Animals

Six adult Sprague Dawley rats (3 males and 3 females, 3 month-old), weighing more than 250g, were provided by the Laboratory of Experimental Surgery of the Medical School of the National and Kapodistrian University of Athens. The rats grew up in stable conditions of heating, ventilation and relative humidity, while their health was daily monitored by a specialized veterinarian. The study was approved by the Directorate of Agricultural and Veterinary Policy and Laboratory of Experimental Surgery (protocol number 1181/2-03-2017, registration code EL25 BIO05, Athens, Greece) and its protocol was in accordance with EU Directive 2010/63/EU, based on the concept of replacement, reduction and refinement of animal studies (“the 3R principle”) (21).

Sample size was minimized in accordance with the aims of “Animal Research: Reporting of *In Vivo* Experimental guidelines (ARRIVE)”. The sample size was estimated with a power analysis (Power 1-β err prob=0.6949) using one-way ANOVA Fixed effects (IBM SPSS 25.0, IBM Corp., Armonk, N.Y., USA). The introduction of the gender as a variable took place in order to limit the use of only male rats, in agreement with the National Institute of Health (NIH, NOT-OD-102). Study groups included two rats (1 male, 1 female) for each time period until euthanasia (2, 4 and 8 weeks, respectively). The number of animals used was comparable to that of previous bone regeneration studies (21).

Bilateral CSDs were made in each rat: the right CSD represented the experimental group, loaded with nHAp/CS scaffold; the left CSD was left empty of biomaterial as the control group. Thus, in total, 12 CSDs were made.

- Surgical procedure

**Figure 1:**
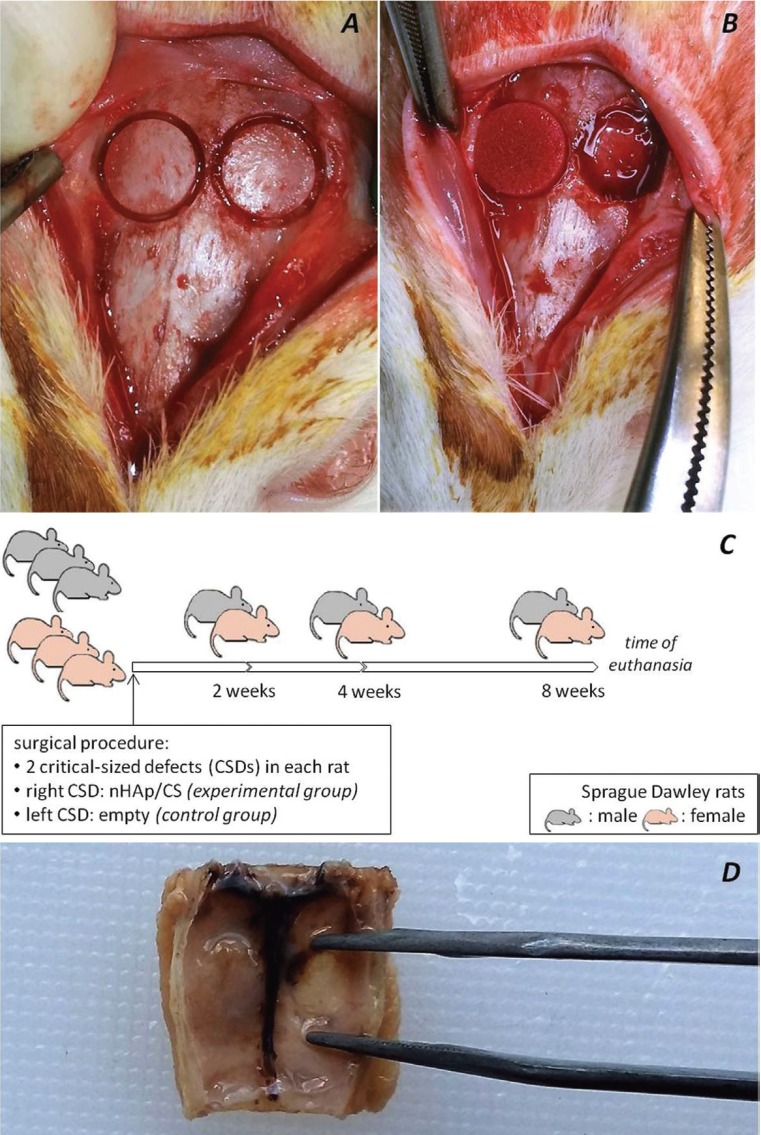
In the dorsal part of the right and the left parietal bones (A) two symmetrical round bone defects were made using a dental drill with a 5mm diameter dental trephine burr and (B) the biomaterial nHAp/CS was placed in the right defect. (C) A timeline diagram showing the distribution of the rats into the groups. (D) Ηistological sections of 5μm were prepared from the mid-point of the defects in the coronal plane.

General anesthesia was induced by intramuscular injection of xylazine 5mg/kg (Rompun, Bayer Animal Health, Leverkusen, Germany) and ketamine hydrochloride 100mg(kg (Imalgene 1000, Merial, Lyon, France). After shaving and disinfecting with povidone-iodine (Betadine Solution, Lavipharm, Athens, Greece) a 2cm-sized longitudinal mid-sagittal skin incision was made in the scalp. The musculature and the mucoperiosteum were exposed and dissected (22,23). Using a dental drill (Implantmed, W&H, Bürmoos, Austria) with a 5 mm diameter dental trephine burr (MT-00500, MIS, Savion, Israel), two symmetrical round bone defects were made in the dorsal part of the right and the left parietal bones (Fig. 1A), under sterile saline irrigation (Sodium Chloride 0.9% Intravenous Infusion, BIOSER, Greece). nHAp/CS was placed in the right CSD (Fig. 1B), while the left CSD was left empty. The wound was sutured in layers with 3-0 polyglactin 910 (Coated VICRYL, Ethicon, Johnson&Johnson, USA). Postoperative care included antimicrobial treatment by intramuscular injection with enrofloxacin 2.5mg/kg (Baytril 5%, Bayer Animal Health GmbH D-51368, Leverkusen, Germany) and analgesic and anti-inflammatory treatment with caprofen (Rimadyl, Pfizer, USA) one time per day, for five days.

Rats were euthanized with diethyl ether (Sigma Aldrich, USA) inhalation at 2, 4 and 8 weeks, respectively (Fig. 1C), after surgical procedure. Calvaria of rats were excised using a surgical sawmill, and two horizontal and two vertical osteotomies were performed that included both the parietal bones and parts of occipital and frontal bones.

- Histological analysis

Specimens were immediately fixed in 10% neutral buffered formalin (Formaldehyde solution, Sigma Aldrich, USA) for one day and then decalcified in EDTA-based solution (MicroDec, Diapath, Italy) for seven days. Histological sections of 5μm were prepared from the mid-point of the defects in the coronal plane (Fig. 1D) and were stained with hematoxylin-eosin (H&E) (23).

- Histomorphometrical analysis

**Figure 2:**
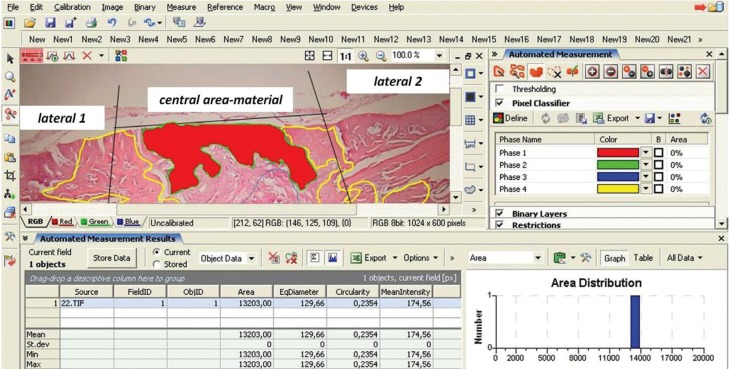
A digital drawing and crop tool separated the area of interest from the near environment and at the final stage digital analysis was performed and area measurements were reported as square px/μm (μm2).

Histomorphometric assessment was performed by a certified digital image analyzer in cytomorphology, who was blind to the intervention. Digital image analysis required a semi-automated system with Intel Pentium V, Digital Camera Sony 1600×1200 and Microscope Olympus CX-31 hardware features, and the following software: Windows XP/Windows XP/NIS-Elements Software AR v3.0, Nikon Corp, Tokyo, Japan. Parameters assessed were the new bone formation in CSDs expressed as μm2 and the NOex-c, defined as total number of osteocytes (OST) in newly formed bone surface in experimental group [experimental OST] minus the total number of osteocytes in newly formed bone surface in control group [control OST]. This number may be considered as a surrogate marker of bone formation speed, as the more rapid the bone formation the more osteocytes are present. For those purposes, division of the corresponding histological sections was implemented by splitting the whole image in continuous areas. A digital drawing and crop tool separated the area of interest from the near environment and at the final stage digital analysis was performed (Fig. 2).

As bone regeneration process from the edge towards the center of the defect, bone formation was evaluated separately on the central and two lateral areas of the defect (22,23), defined as Regions of Interest (ROI), as follows: (a) lateral area inward of the middle sagittal seam (late-ral 1, l1); (b) lateral area outward of the middle sagittal seam (lateral 2, l2) and (c) central area (c, central) (Fig. 2). New bone surface and NOex-c were calculated for each ROI in H&E-stained sections with original magnifications x100.

- Statistical analysis

The data, i.e. new bone surface as μm2 and total number of osteocytes, were estimated as mean±Standard Deviation (SD). The calculation of the sample size was made in accordance with the aims of ARRIVE guidelines, based on previous literature (21). A one–way ANOVA and Student’s paired t-test using IBM SPSS 25.0 were applied to determine possible significant differences, considering a level of significance of *P*<0.05.

## Results

- Animals

During and after surgical procedure, no visible complication was seen in any experimental animal. Wound healing was excellent in all rats.

- Histological results

Homogeneity of the size of all the CSDs was evaluated by statistical analysis. The total surface of all CSDs (in μm2) were similar, as there were no statistically significant differences between experimental and control groups (*P*>0.05), in relation to sex (*P*>0.05) and to the euthanasia time (2, 4, 8 weeks) (*P*>0.05).

12 CSDs were available for microscopic evaluation: the experimental group, consisting of 6 CSDs on the right side, where nHAp/CS scaffolds were placed; and the control group, consisting of 6 CSDs on the left side that was left empty of the nHAp/CS scaffolds.

Newly formed bone appeared as fibrous bone with many osteocytic lacunae. Therefore it could be easily distinguished by the preexisting calvarial bone at the edges of the CSDs that was lamellar and sparsely cellular. Residual graft material was observed in the CDSs of all rats in the experimental group, but due to the small sample size no effort to quantify it in relation to time interval was made. Scaffolds of nHAp/CS showed good integration at 2, 4 and 8 weeks, as shown by lack of pus formation, necrosis, or foreign body granuloma formation in any of the CSD of the experimental or control group ( Table 1).

**Table 1:**
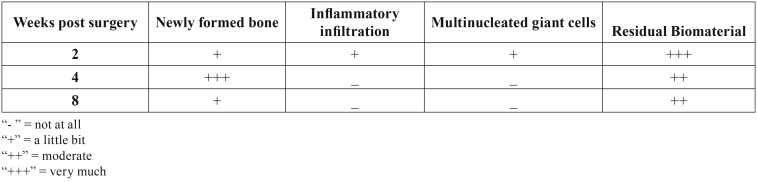
Histological parameters evaluated at 2, 4 and 8 weeks post surgery.

**Figure 3:**
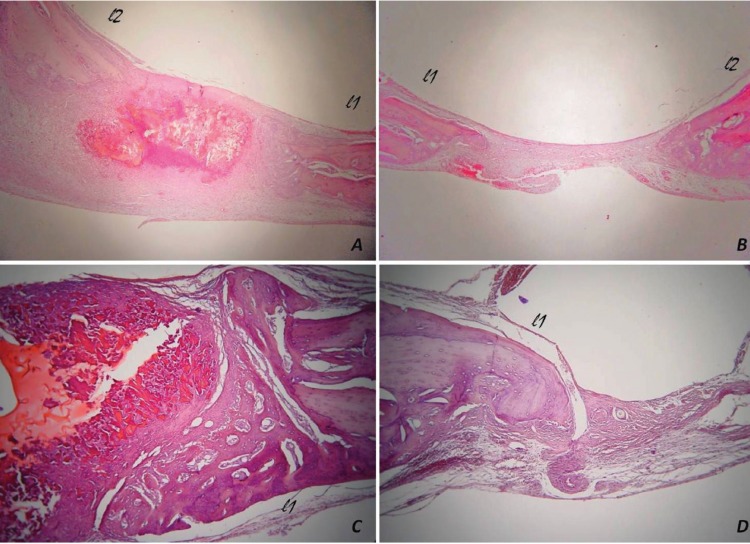
In rats euthanized at 2 weeks post surgery, newly formed bone was observed in the lateral areas inward (lateral 1, l1) and outward (lateral 2, l2) of the middle sagittal seam, while not in the central area of (A) experimental and (B) control group. In rats euthanized at 4 weeks after surgery, the number of osteocytes in new bone area was higher in (C) the experimental group, compared with (D) the control group [hematoxylin and eosin stain, original magnifications (A, B) x25, (C, D) x100].

In rats euthanized at 2 weeks after surgery, some newly formed bone was observed on the l1 and l2 areas of both experimental and control group, but not in the central area (Fig. 3A, B). Mild inflammatory infiltration and few osteoclast-like, multinucleated giant cells were observed around the biomaterial.

In rats euthanized at 4 weeks after surgical procedure, more newly formed bone was observed on the l1 and l2 areas of the experimental and the control group. Newly bone formation in the central area of CSD with the biomaterial was observed in the female rat (Fig. 3C, D). There was no inflammatory infiltration or multinucleated giant cells in relation to the biomaterial.

In rats euthanized at 8 weeks after surgery, newly formed bone was observed on the l1 and l2 areas of both groups. Bone formation was also found in the central area of the unfilled CSD in the male rat. There was no inflammatory infiltration or multinucleated giant cells in relation to the biomaterial.

There were no statistically significant differences among the animals in relation to the gender.

- Histomorphometrical results

In  Table 2, the mean surface of newly formed bone (in μm2) for ROIs of each animal was presented. There was a statistically significantly increase in the surface of newly formed bone in l1 (*P*=0.039) in the experimental group (91733.00±38855.60 μm2) compared to the control group (46762.17±25507.97 μm2) ( Table 2). Within the experimental group, a statistically significant increase (*P*=0.042) in the surface of newly formed bone in l1 area was noticed in rats euthanized in 4th week compared with rats euthanized in 2nd week after surgery ( Table 3).

**Table 2:**
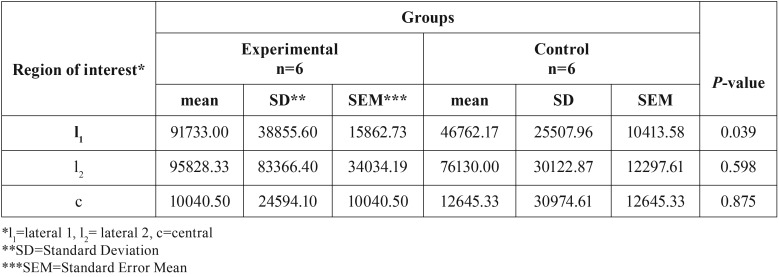
Surface of newly formed bone (in μm2) in lateral (l1, l2) and central (c) regions of interest in experimental and control group of all rats.

**Table 3:**
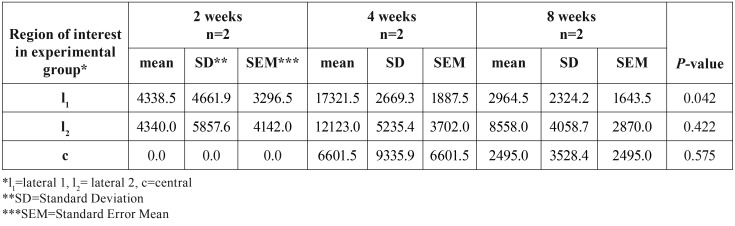
Newly bone formation (in μm2) in the experimental group in rats euthanized at 2nd, 4th and 8th week after surgery.

In  Table 4, the NOex-c in 2, 4 and 8 weeks after surgery is also presented. Τhere was a statistically significant increase (*P*=0.029) in this parameter in rats euthanized in 4th week post surgery ( Table 4).

**Table 4:**
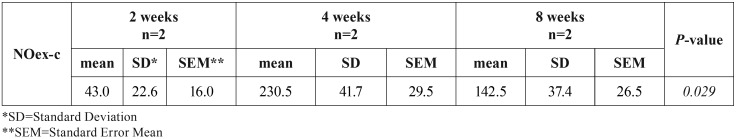
NOex-c, i.e. total number of osteocytes in newly formed bone surface in experimental group minus total number of osteocytes in newly formed bone surface in control group, in rats euthanized at 2nd, 4th and 8th week after surgery.

There were no statistically significant differences among the animals in relation to the gender.

## Discussion

In the present study new bone formation, as assessed by newly formed bone surface and total number of osteocytes, was seen in bilateral CSDs of 5mm-diameter in Sprague Dawley rats filled with 75/25 w/w nHAp/CS scaffolds.

CSD is “the smallest intrabony wound which does not heal spontaneously” (24). The proper CSD size for evaluation of bone regenerative biomaterials in a rat calvarial defect model has not been determined and full-thickness CSDs of 5mm-diameter (21,23,25-29) or 8mm-diameter (22,30-32) have been used. Full-thickness 5mm-diameter defects simplify surgical procedure and allow the creation of bilateral defects, reducing rats’ mortality and morbidity (21,33), and consequently the total number of animals that are required (21). This experimental setting allowed the comparative histological and histomorphometric analysis of the defects in the same animal in the current study. However, full-thickness 5mm-diameter unfilled defects may show bone formation at 8 weeks (34), as seen in the male rat euthanized at 8 weeks after surgery in the current study.

Histomorphometrical analysis is the gold standard for the assessment of new bone formation in rat calvarial CSDs (21,22,29). In this study, digital image analysis allowed an objective comparison of new bone formation among experimental and control CSDs, as estimated by the surface of the newly formed bone and number of osteocytes.

The choice of both sexes in this study was made in agreement with the National Institute of Health (NIH) “Consideration of Sex as a Biological Variable in NIH-funded Research” notice (NOT-OD-102) that has marked an over-reliance on male animals and cells in global research. The use of both sexes also eliminates gender as a variable in the statistical analysis (32). No statistically significant difference in new bone formation surface was seen between male and female rats in our study. Howe-ver, as the number of animals used was small, more studies are necessary to confirm this finding.

A study comparing micro-Hydroxyapatite/Chitosan (mHAp/CS) and nHAp/CS scaffolds showed that the total volume, bone volume, bone density and bone surface was higher in nHAp/CS compared with mHAP/CS scaffolds (35). Porous scaffolds based on nHAp/CS are commonly used in tissue engineering applications. Those scaffolds have a highly porous structure with pore size ranging from 200μm to 700μm (17,22,28,36), when the optimal size for bone ingrowth is 100μm to 400μm (37-41). In previous studies investigating the biological behavior of 80/20 w/w of nHAp/CS in rats, it was observed that when the hydroxyapatite concentration exceeded 80% the resulting scaffolds were friable (17). Therefore, Tsiourvas *et al.* (17) proposed that a ratio of nHAp/CS of 75/25 w/w with 90% porosity provided the scaffolds with improved physico-mechanical properties. In the present study a nHAp/CS of 75/25 w/w with 85±2% porosity, with pore size ranging between 20-100μm, was used and our findings support this proposal also from a “clinical” point of view, as during surgical procedure, the biomaterial was more easily cropped, handled, stuffed and covered by the tissue flaps in the CSDs.

Considering new bone formation, a statistically significant increase was seen in the surface of the bone formed in the lateral regions of the defects between 2nd and 4th week. Those results are in agreement with previous studies (16,22,28,36). In addition, in the experimental group, a statistically significant increase was observed in the surface of newly formed bone in rats euthanized in 4th week compared to those euthanized in 2nd week after surgery, but not between the 4th and 8th week or the 2nd and 8th week. Overall, the number of animals in each group was small for definite conclusions to be drawn.

In conclusion, scaffolds of nHAp/CS integrate bone tissue and provide an effective space for new bone formation. However, those results are not necessarily applicable to alveolar bone. Thus, further investigation of nHAp/CS scaffolds for GBR in oral and periodontal reconstructive procedures is required.
